# Metabolomic Profiling of Seasonal Katsumadin Production in *Ternstroemia lineata*

**DOI:** 10.3390/molecules31060964

**Published:** 2026-03-13

**Authors:** Alexis Uriel Soto-Díaz, María Luisa Villarreal, Nahim Salgado Medrano, Marcelo Victorio-De los Santos, Edda Sciutto, José Alejandro Espinosa Cerón, Jan Schripsema, Alexandre Toshirrico Cardoso-Taketa

**Affiliations:** 1 Centro de Investigación en Biotecnología, Universidad Autónoma del Estado de Morelos, Cuernavaca 62210, Mexico; uriel.soto.diaz@outlook.com (A.U.S.-D.); luisav@uaem.mx (M.L.V.); 2 Facultad de Ciencias Biológicas, Universidad Autónoma del Estado de Morelos, Cuernavaca 62210, Mexico; nahim@uaem.mx; 3 Unidad Académica de Ciencias Químicas Biológicas y Farmacéuticas, Universidad Autónoma del Nayarit, Nayarit 63000, Mexico; mvictorio@uan.edu.mx; 4 Departamento de Inmunología, Instituto de Investigaciones Biomédicas, Universidad Nacional Autónoma de México, Ciudad de México 04510, Mexico; edda@unam.mx (E.S.); alexec2803@gmail.com (J.A.E.C.); 5 Grupo Metabolômica, Universidade Estadual do Norte Fluminense Darcy Ribeiro, Campos dos Goytacazes 28013-602, RJ, Brazil

**Keywords:** *Ternstroemia lineata* DC, katsumadin, HPLC, ^1^H NMR, metabolomics, PCA

## Abstract

Metabolomic profiling of the leaves, floral buds, flowers, and fruits of *Ternstroemia lineata* was conducted across the dry and rainy seasons in Mexico. The presence of katsumadin was determined and its antibacterial activity was evaluated. Multivariate data analysis was performed using proton nuclear magnetic resonance (^1^H NMR) and Principal Component Analysis (PCA). Katsumadin was identified by ^1^H NMR and, quantified by HPLC, and its antibacterial activity was assessed using a microdilution assay. The results revealed conserved ^1^H NMR signals among leaf, flower buds, flowers and fruits, as well as signals that were either organ-specific or time-dependent. The spatial and temporal distribution of katsumadin was monitored over a nine-month period. During the dry season, katsumadin reached 1.5 mg/g DW in the leaves, whereas it was not detected during the rainy season. Higher katsumadin contents were observed in floral buds, fruits, and flowers (6.09 ± 0.10, 3.0 ± 0.79, and 3.4 ± 0.42 mg/g DW, respectively). In addition, katsumadin was evaluated against *Salmonella* Typhi and *Pseudomonas aeruginosa*, exhibiting a minimum inhibitory concentration and bactericidal effect at 0.5 mg/mL.

## 1. Introduction

The genus *Ternstroemia* (Pentaphylacaceae) comprises approximately one hundred species worldwide, ten of which are distributed in Mexico. *T. lineata* is native to Mexico and Central America, where it inhabits cloud forests and other humid environments [1,2]. Infusions prepared from the leaves and flowers have been used in Mexican traditional medicine for the treatment of various ailments, including gastric, respiratory, and cutaneous infections [3].

Among plant secondary metabolites, phenolic compounds such as phenylpropanoids constitute a chemically diverse group with well documented antioxidant and antibacterial activities [4,5]. Our research group has previously described the antioxidant and antibacterial activities of the *T. lineata* extracts [5,6,7]. The biphenyl propanoid katsumadin has been reported in *T. lineata* fruits with high antioxidant activity (73.93 ± 7.1 µM) in the ABTS•+ model and protected the *Saccharomyces cerevisiae* cells from H_2_O_2_-oxidative stress at 25 µM [6,7]. The antibacterial activity of the hydroalcoholic and phenolic leaf extracts of *T. lineata* has been evaluated against a panel of Gram-negative bacteria, showing activity against *Salmonella enterica* subsp. *enterica* serovar Typhi (*S.* Typhi) and *Pseudomonas aeruginosa*, with minimum inhibitory concentration (MIC) values of 6.0 and 4.0 mg/mL, respectively [5]. These findings indicate that phenolic compounds contribute to the observed biological activity.

Numerous plant-derived phenylpropanoids exhibit both antioxidant and antibacterial activities [8]. These compounds can disrupt cell membrane integrity, interfere with key enzymatic processes, and induce oxidative stress by disturbing cellular redox balance [9,10]. The presence of the phenylpropanoid katsumadin in the leaves of *T. lineata* raises questions about its antibacterial potential. This secondary metabolite has been reported previously in the fruits of the plant [7]. As fruits are produced only once annually, the leaves of *T. lineata* offer an alternative for the exploration and potential utilization of katsumadin.

The spatial and temporal distribution of plant secondary metabolites may respond to ecological interactions and plant adaptation, and their synthesis is often strongly influenced by seasonal environmental variation [11]. Changes in rainfall patterns, temperature, and water availability can induce substantial shifts in plant metabolism, leading to temporal fluctuations in the synthesis and accumulation of metabolites [12]. Phenylpropanoids represent one of the classes of secondary metabolites most responsive to environmental changes. Water limitation during dry seasons has been suggested to influence the accumulation of phenylpropanoids in plants, some of which exhibited antibacterial properties [13,14].

An integrative analysis of changes across floral developmental stages and fruits, together with vegetative tissues such as leaves and temporal factors, can provide valuable insights into the distribution and concentration of katsumadin in *T. lineata.* Metabolomic approaches, particularly those combining untargeted profiling with compound annotation, have emerged as powerful tools for characterizing plant chemical diversity. By integrating advanced analytical techniques such as ^1^H NMR spectroscopy, this study employs a metabolomic strategy to investigate the spatial and temporal distribution of phenolic compounds, such as the phenylpropanoid katsumadin, in *T. lineata* leaves and flowers at different developmental stages, with the aim of elucidating its organ-specific occurrence and potential biological significance. There is currently a lack of information regarding how the accumulation of katsumadin varies among different plant organs, developmental stages, and seasonal conditions in *T. lineata.* Furthermore, its potential ecological relevance remains poorly understood. Therefore, the novelty of the present study lies in providing the first integrative metabolomic characterization of distribution of phenolic compounds in *T. lineata,* with special emphasis on katsumadin. We hypothesize that katsumadin accumulation is different organs and development dependent and may increase during the dry season, potentially reflecting an adaptative metabolic response to seasonal environmental conditions.

## 2. Results

### 2.1. Metabolomic Variation in Leaves and Floral Stages

Metabolic profiling of *T. lineata* leaves revealed time-dependent changes based on ^1^H NMR spectra. Overall aromatic content was higher between December–April, compared to June and August. Aromatic compounds reached their maximum concentration in winter (December–February), followed by a decrease in spring to 73.84%, with a further reduction to 46.00% during the rainy season (June–August).

A similar pattern of variation was observed for carbohydrate content, with higher number of signals between December and April compared to June and August, when they clearly decreased.

**Figure 1:**
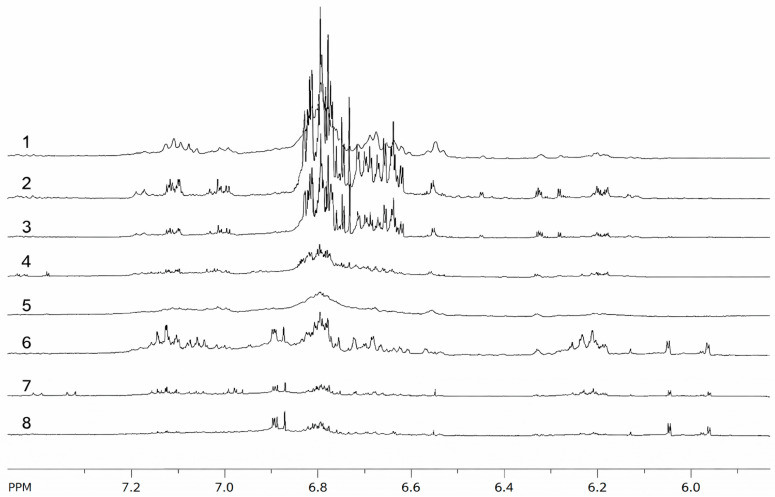
Comparison of the aromatic regions in the ^1^H NMR spectra of the hydroalcoholic extract from leaves collected in December (1), February (2), April (3), June (4), August (5), floral buds (6), flowers (7), and fruits (8). TMSP signal was used to normalize the signal intensities.

Among the different floral developmental stages, the floral buds exhibited the highest number of signals in the aromatic region of the spectra, which decreased after the bud transitioned into flowers, which subsequently developed into fruits (Figure 1).

**Figure 2:**
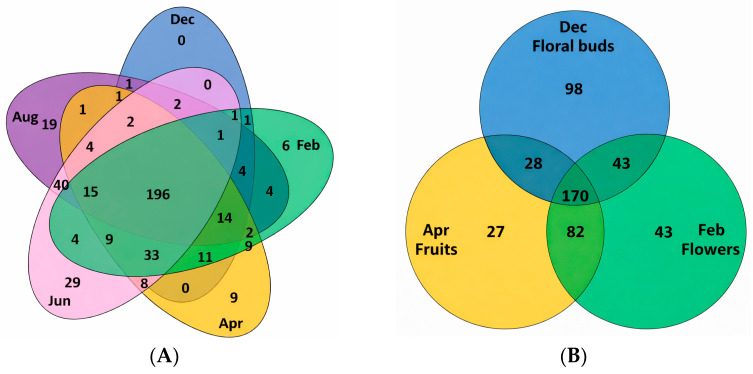
Venn diagram of five-sets for leaves (**A**) and three-sets for floral stages and fruits (**B**) from the hydroalcoholic ^1^H NMR spectra.

A five-set Venn diagram was constructed using the signals recorded in the ^1^H NMR resonance spectra from leaf hydroalcoholic extracts (Figure 2A and Table S1). A total of 263, 310, 313, 346, and 303 signals were detected in Dec, Feb, Apr, Jun, and Aug, respectively. Overall, 424 distinct signals were observed in the leaf profiles over the study period, of which 196 were present in all months, corresponding to 46.2% of the total signals detected in the ^1^H NMR spectra. Some of these shared signals correspond to essential primary metabolites of leaves, such as carbohydrates (sucrose, glucose, and fructose), amino acids (threonine, alanine, and valine), and organic acids (citric, acetic, malonic, and formic acids), Table S2 [15,16,17]. Each month, except December, exhibited unique signals that were not observed in other months, with June showing the highest number of unique signals (29). Figure 2B presents a three-set Venn diagram based on the signals observed in the ^1^H NMR spectra of floral stages and fruits. A total of 339, 338, and 307 signals were detected in the spectra of floral buds, flowers, and fruits, respectively. In total 491 signals were observed in the floral stages and fruits’ resonance profiles, of which only 170 were presented in each stage, corresponding to 34.6% of the total signals visualized. Each month presented unique signals that were not observed in other stages, being floral buds the period with higher amount (98), followed by flowers (43) and fruits (27).

### 2.2. Identification of Metabolites in the Leaves, Floral Stages, and Fruits by ^1^H NMR

Hydroalcoholic extracts were analyzed by ^1^H NMR spectroscopy to identify metabolites and to assess the metabolic variation over time. The proton spectra enabled the identification of metabolites such as sucrose (δ 5.40, d, *J* = 3.97), α-glucose (δ 5.20, d, *J* = 3.66), β-glucose (δ 4.6, d, *J* = 7.93), and fructose (δ 3.90, dd, *J* = 9.9, 3.4) [15,16]. The high-field region of the spectra contained signals corresponding to amino and organic acids such as valine (δ 0.95, d, *J* = 6.71; δ 1.02, d, *J* = 6.1), threonine (δ 1.32, d, *J* = 6.41) and alanine (δ 1.49, d, *J* = 7.32) [15,16,17]. Citric acid was identified by characteristic chemical shifts at δ 2.56 (H-2) and δ 2.73 (H-4); while malonic acid was identified by a signal at δ 3.11 (H-3) [16,17]. Acetic acid was identified by its methyl group signal at δ 1.90. Succinic acid was identified by a single high-field signal at δ 2.39 (s). In the floral stages, all of these metabolites were also detected, along with characteristic signals of γ-aminobutyric acid (GABA), a common metabolite in many plant species.

In addition, the ^1^H NMR spectrum of the neurotoxic metabolite 28-O-[β-L-6-rhamnopyranosyl]-barrigenol previously reported in the seeds of *T. sylvatica* [18] was compared with the spectra of the extracts from the floral stages and fruits, including seeds, and no corresponding signals of the saponin were detected in any extract.

^1^H NMR signals of katsumadin were observed in leaves (Dec, Feb, and Apr), floral stages, and fruits of *T. lineata.* The structure of this biphenylpropanoid (C_15_O_6_H_14_), composed of two benzene rings tetra and trisubstituted, was identified by ^1^H NMR spectra through the signal pattern corresponding to an ABX system for the aromatic proton H-6′, H-5′ and H-2′ (Table 1). The tetrasubstituted ring was characterized by the presence of two doublets at δ 5.87 (H-4″, d, 2.3 Hz) and δ 5.94 (H-6″, d, 2.3 Hz), corresponding to a meta-coupled spin pattern.

### 2.3. Quantification of Katsumadin in Leaf, Floral Stages, and Fruit Hydroalcoholic Extracts by HPLC

**Figure 3:**
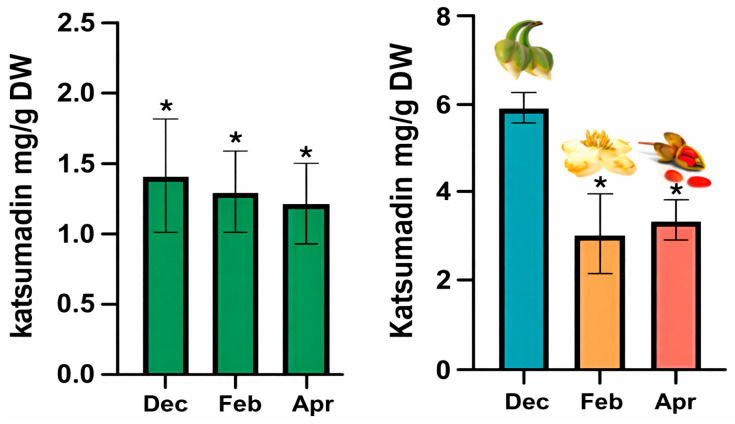
Left. Katsumadin concentration in leaves (Dec–Apr); Right. Floral buds (blue), flowers (orange), and fruits (pink). Data are presented as mean ± SD (*n* = 3) and statistical analysis was performed using one-way ANOVA followed by Tukey’s post hoc test. Asterisks indicate no statistically significant differences (*p* < 0.05).

Purified katsumadin [7] was used as a standard to quantify this metabolite in hydroalcoholic extracts of leaves, floral buds, flowers, and fruits. The compound eluted with a retention time of 8.55 min in the HPLC analysis. Figure 3 shows the concentration of katsumadin relative to the dry weight (DW) of the extracts. Katsumadin was detected only in leaf extracts from December, February, and April, at concentrations of 1.40 ± 0.36, 1.29 ± 0.25 and 1.20 ± 0.24 mg/g DW, respectively. No statistically significant differences were observed in these months. Notably, katsumadin was not detected in June and August, which correspond to the rainy season in Mexico.

Climatic data obtained from the meteorological station of the National Water Commission (CONAGUA), corresponding to the study area, showed marked seasonal variation in precipitation, evaporation, and temperature during the sampling period. The months from December to April corresponded to the dry season and were characterized by minimal precipitation, with recorded rainfall ranging from 0 to 48.5 mm. In contrast, June and August corresponded to the rainy season, with substantially higher precipitation levels reaching 163.7 and 394 mm, respectively. Total monthly evaporation values ranged between 80.94 and 142.64 mm throughout the study period. During the dry season, precipitation was absent or scarce, whereas evaporation remained between 80.94 and 121.26 mm. In April, although precipitation increased to 48.5 mm, evaporation reached 142.64 mm. Temperature records also showed seasonal variation, with maximum temperatures ranging from 19 to 24 °C and minimum temperatures from −2 to 6 °C. Temperature progressively increased toward the rainy season, reaching 13.2 °C in August.

This is the first report of the presence of katsumadin in the leaves of *T. lineata*, revealing a temporal distribution in this organ. In contrast, other organs displayed higher katsumadin concentrations: floral buds (6.09 ± 0.10 mg/g DW) collected in December, flowers (3.0 ± 0.79 mg/g DW) collected in February, and fruits (3.4 ± 0.42 mg/g DW) collected in April.

### 2.4. Principal Component Analysis

**Figure 4:**
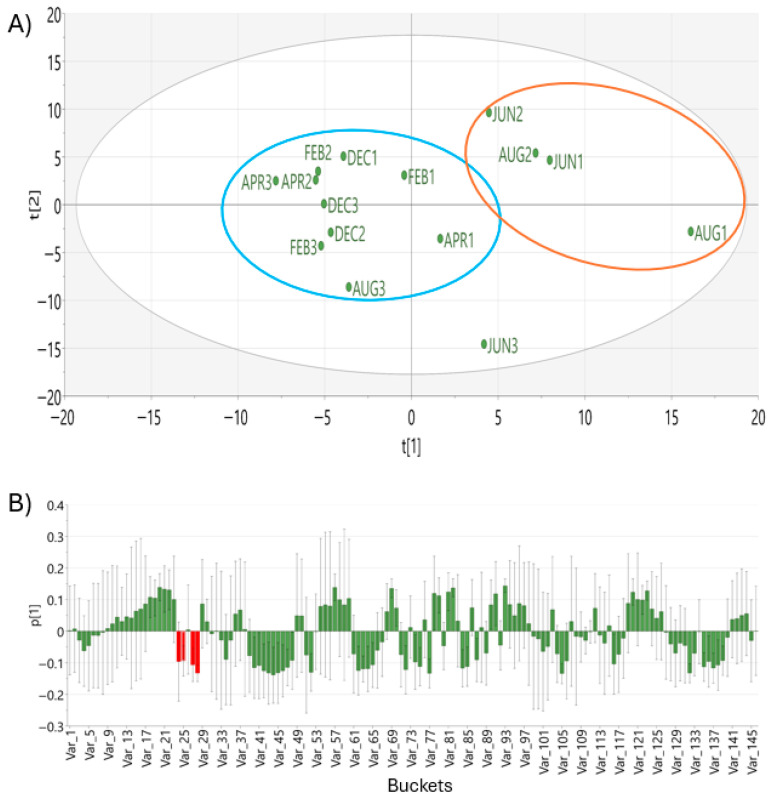
(**A**) PCA score plot of leaf samples collected in different months; (**B**) Bar loading of PC1 from PCA. The red bars represent the loading coefficient of katsumadin, indicating its contribution to the observed separation between the samples. Blue ellipse corresponds majority to the months of dry season (December to April) and the red ellipse to the rainy season (August and June). The variables, Var_1 to Var_145 were obtained from the bucketing of the 1H NMR spectra at 0.05 ppm.

Multivariate data analysis of leaf hydroalcoholic extracts collected in different months was performed using ^1^H NMR spectra and principal component analysis (PCA). The score plot from the unsupervised analysis revealed a clear metabolic difference among the extracts (Figure 4A). The PCA model with two components showed a cumulative explained variance of R^2^X = 0.575, indicating that 57.5% of the total variability in the dataset was captured by the model. The predictive ability of the model, estimated by cross-validation, yielded a Q^2^ value of 0.31, suggesting moderate predictive relevance. Since biological replicates influence Q^2^, a higher number of samples per baches is recommended for further studies. drive Q^2^. The DModX plot was used to evaluate the goodness of fit of individual samples within the PCA model. All observations remained below the critical limit (DCrit [0.05] = 1.33), indicating that none of the samples exceeded the 95% confidence threshold (Figures S1 and S2). This finding confirms the absence of moderate or strong outliers and supports the robustness of the PCA model.

Variables with the highest absolute loadings in PC1 were associated with signals located at δ 5.85, 5.90, 6.75 and 6.85, corresponding to aromatic resonances of katsumadin. These results suggest that this metabolite contributes to seasonal discrimination, even though other metabolites are also involved. The first principal component (PC1) grouped most of the extracts corresponding to June and August from those obtained in December, February and April, suggesting metabolic variation associated with sampling time. The second principal component (PC-2) further highlighted the distinction between samples collected during the rainy and dry seasons. Overall, the extracts tended to cluster according to sampling months, indicating that temporal variation exerts a major influence on the metabolic profile of plants collected in different seasons.

**Figure 5:**
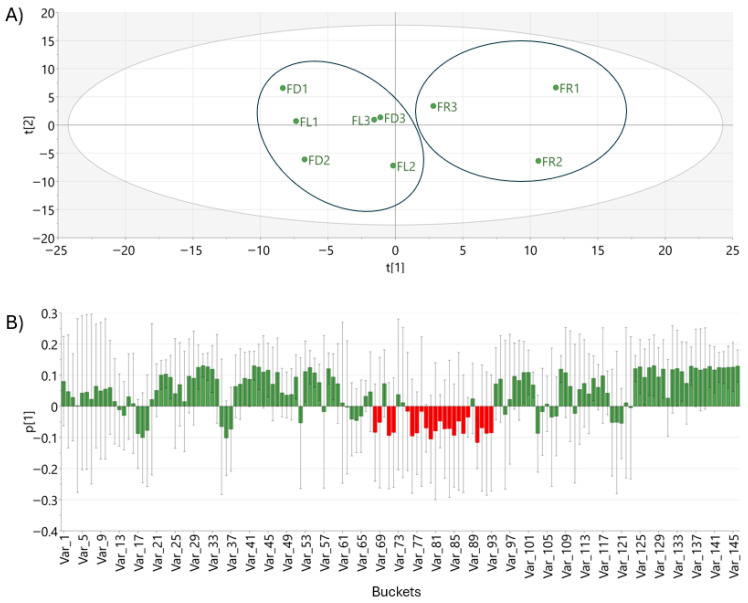
(**A**) PCA score plot of leaf samples collected in different months; (**B**) PC-1 score plot corresponding to floral buds (FD); flowers (FL), and fruits (FR). The red bars represent the loading coefficient of sugar region, indicating its contribution to the observed separation between the samples.

The PCA of extracts, corresponding to different plant developmental stages: flower buds (FD) in December, flowers (FL) in February, and fruits (FR) in April, revealed clear metabolic differentiation among samples (Figure 5A). PC1 explained 37.2% of the total variability, while PC2 accounted for 19.9%, together explaining 57.1% of the total variance. Along with PC1, a clear separation was observed between extracts from April (fruits) and those from December (flower buds) and February (flowers), indicating that the primary source of metabolic variation is associated with the developmental stage, which is accompanied by seasonal progression.

Overall, the PCA score plot indicates that the metabolic profiles of the extracts are strongly influenced by both the developmental stage and the sampling period (Figure 5A). The observed clustering pattern reflects metabolic changes associated with the transition from flower buds to flowers and fruits over the analyzed months. The loading plot (red bars) of PC-1 revealed that signals located in the sugar region of the NMR spectra exerted a strong influence on group separation, especially differentiating fruits from the other organs (Figure 5B). The predictive ability of the model, estimated by cross-validation, yielded a Q^2^ value of 0.17, suggesting moderate-low predictive relevance, due to high biological variability. All samples presented DModX values below the critical limit (DCrit = 1.413, α = 0.05), indicating that no strong outliers were detected and that all observations are well described by the model (Figure S2). These results confirm the robustness and structural consistency of the PCA model.

### 2.5. Antibacterial Activity of Katsumadin

Both the minimum inhibitory concentration (MIC) and minimum bactericidal concentration (MBC) of katsumadin against *S.* Typhi and *P. aeruginosa* were determined (Table 2). Katsumadin inhibited the growth of both Gram-negative bacteria at a concentration of 0.5 mg/mL. To further evaluate its bactericidal effect, aliquots from each microplate were centrifuged, washed and subcultured on Mueller-Hinton (MH) agar. At a concentration of 0.5 mg/mL, katsumadin completely inhibited bacterial growth, and no colony-forming units (CFUs) were detected. These results indicate that this biphenylpropanoid exhibits a bactericidal effect against the tested bacteria.

## 3. Discussion

The metabolomic profiles obtained by ^1^H NMR and HPLC analyses clearly demonstrate pronounced temporal variation in the secondary metabolism of *T. lineata* during the study period, particularly in leaf tissues. A higher abundance of aromatic compounds during the dry season (December–April), followed by a marked decrease with the onset of the rainy season, suggests that secondary metabolite biosynthesis in leaves is tightly regulated by seasonal environmental factors. Aromatic compounds, including phenylpropanoids, are frequently associated with plant defense responses and stress adaptation, and their accumulation under dry conditions may reflect an adaptive strategy [19,20].

The seasonal pattern observed in carbohydrate content further supports the concept of coordinated metabolic regulation. During dry periods, altered carbon allocation may favor the synthesis of secondary metabolites derived from primary metabolic pathways, whereas during the rainy season, metabolic flux appears to shift toward growth-related processes. In tropical and subtropical ecosystems, such as Mexico, where winter coincides with the dry season, long periods of light exposure, even when total radiation is not maximal, may stimulate the photosynthesis activity. In this scenario, sugar synthesis is increased, and the metabolic reorientation toward the production of secondary metabolites, such as phenolic derivatives, takes place, taking advantage of the excess reducing power (NADPH) generated by photoreduction [21,22,23]. Climatological data obtained from CONAGUA corresponding to the collection area indicate that December to April was characterized by minimal precipitation (0–48.5 mm total rainfall) and low mean rainfall values (0–1.6 mm), whereas June and August presented substantially higher precipitation levels (163.7 and 394 mm total rainfall, respectively). In addition, evaporation during dry months (80.94–142.64 mm total evaporation) was comparable to or exceeded total precipitation, reinforcing the presence of water deficit conditions during this period. The combination of negligible rainfall and sustained evaporation during the dry season suggests persistent hydric stress, which may act as an environmental driver promoting enhanced phenylpropanoid pathway activity. In contrast, the rainy season exhibited a clear shift toward positive water balance, with precipitation markedly surpassing evaporation, potentially reducing the need for stress-associated secondary metabolite production. Water limitation is one of the most important abiotic stress factors influencing plant metabolism [14].

Across the floral developmental stages, including fruits, the relatively low proportion of shared ^1^H NMR signals indicates a high degree of metabolic specialization associated with reproductive development. Floral buds exhibited the highest number of unique signals, suggesting intense metabolic activity during early reproductive stages, likely related to tissue differentiation or protection of developing reproductive organs. The gradual decrease in the number of unique metabolites from buds to fruits is consistent with a metabolic streamlining process as reproductive development progresses [24,25].

The identification of primary metabolites such as carbohydrates, amino acids, and organic acids in both leaves and floral tissues reflects conserved core metabolic processes across organs and developmental stages. The detection of GABA in floral stages further supports the involvement of stress-related and signaling pathways during reproductive development, as GABA is known to play roles in plant stress responses and developmental regulation [26].

The identification of katsumadin in leaves, floral developmental stages, and fruits represents a significant contribution to the phytochemical knowledge of *T. lineata*. While previous reports restricted its occurrence to fruits [7], the present study demonstrates for the first time that katsumadin is also synthesized and accumulated in leaves, albeit in a strictly seasonal manner. The exclusive detection of katsumadin in leaf extracts during the dry season highlights strong environmental control over its biosynthesis.

Phenylpropanoid-derived compounds are widely recognized as responsive to environmental cues, particularly water availability. The seasonal accumulation of katsumadin during periods of low or absent rainfall may therefore reflect a broader activation of the phenylpropanoid pathway under stress conditions. Although a direct defensive function of katsumadina was not experimentally evaluated in planta, its seasonal accumulation pattern, together with its demonstrated antibacterial activity in vitro, is consistent with a potential ecological role associated with stress responses. However, further functional studies would be required to confirm such a role.

In reproductive tissues, katsumadin exhibited a clear developmental pattern, with the highest concentrations detected in floral buds followed by a progressive decrease in flowers and fruits. This distribution suggests that katsumadin biosynthesis is developmentally regulated and may be related to a protective role during early reproductive stages, when tissues are particularly vulnerable to microbial infection. While phenylpropanoids in general have been implicated in processes such as reproductive development, auxin transport modulation, and tissue differentiation, no direct experimental evidence currently supports a specific role for katsumadin in these processes. Therefore, its higher accumulation in early floral stages may reflect enhanced phenylpropanoid pathway activity during organ differentiation rather than a demonstrated regulatory function [4,9].

Katsumadin was detected in fruits at lower concentrations, indicating its persistence during fruit development. Moreover, because phenylpropanoids are precursors of flavonoids (such as quercetin and kaempferol), that are essential for pollen fertility and pollen tube growth [27,28], the markedly higher production of katsumadin (approximately twofold) in floral buds, compared to other plant organs, is likely biologically justified. In addition, the elevated production of the phenylpropanoid katsumadin in the early floral stages could support previous findings that link this metabolite to the plant growth processes, involving auxin transport, including tissue differentiation and floral development [29,30]. In addition to biosynthetic regulation, variation in katsumadin levels may also reflect differences in metabolite turnover or degradative processes. Seasonal shifts in enzymatic activity could influence the stability, conversion or degradation rate of phenylpropanoid-derived compounds, thereby contributing to the observed fluctuations in relative abundance [14].

Principal component analysis revealed that both sampling time and developmental stages exert a stronger influence on metabolomic profiles than individual variability, underscoring the importance of temporal and developmental factors in shaping plant secondary metabolism. The clear separation between dry-season and rainy-season leaf extracts supports the conclusion that seasonal variation is a primary driver of metabolic differentiation in *T. lineata*.

In floral stages, PCA further highlighted coordinated metabolic changes associated with reproductive development and seasonal progression. Although most detected signals remain unidentified, the strong contribution of katsumadin to the observed clustering patterns indicates that this compound plays a significant role in the metabolic differentiation among samples.

The demonstration of antibacterial activity of katsumadin against *S.* Typhi and *P. aeruginosa* provides direct evidence of its biological relevance. The observed MIC and MBC values (0.5 mg/mL) indicate that katsumadin exerts a bactericidal effect against these Gram-negative pathogens. Our previous reports of *T. lineata* showed antibacterial effect of leaf hydroalcoholic extract and phenolic fraction at 6 and 4 mg/mL, respectively [5]. The present study has limitations, including the absence of direct measurements of enzymatic activity, precursor availability, and metabolite turnover rates, but provides the first quantitative evidence of seasonal variation of katsumadin in *T. lineata* and demonstrates its antibacterial activity against pathogenic bacteria. Our findings establish a direct experimental link between seasonal biosynthesis and antimicrobial activity, by integrating ecological patterns of metabolite accumulation with functional bioassays. These results provide novel insights into the ecological relevance of katsumadin and offer a biochemical basis for the traditional medicinal use of *T. lineata.* The collection and use of the leaves to treat infections may be most effective during the dry season, when katsumadin production is elevated.

Overall, the integration of metabolomic profiling, quantitative analysis, and antibacterial assays reveals katsumadin as a seasonally regulated secondary metabolite with both ecological and pharmacological significance. These findings underscore the importance of considering temporal variation in phytochemical studies and highlight *T. lineata* as a promising source of bioactive compounds with antibacterial potential.

Notably, the absence of ^1^H NMR signals corresponding to the neurotoxic saponin previously reported in *T. sylvatica* seeds suggests a species-specific metabolic profile within the genus *Ternstroemia*. This finding has ecological and pharmacological relevance, as it indicates that the reproductive tissues of *T. lineata*, including fruits with seeds, do not accumulate this potentially harmful compound [18]. Nevertheless, future studies, employing analytical techniques more sensitive than ^1^H NMR are warranted to definitively confirm the absence of this toxic saponin in *T. lineata*.

## 4. Materials and Methods

### 4.1. Plant Material Collection

Leaves from four individuals of *T. lineata* were collected every two months, beginning in December 2016 and continuing until August 2017, in the municipality of Huitzilac, located in the state of Morelos (coordinates 19.026558, −99.274132), Mexico. To reduce variability associated with tissue age, leaf samples were consistently collected from the same height of the trees throughout the study period. Mature leaves without visible signs of senescence, mechanical or herbivory damage were selected at each sampling time. Although seasonal progression inevitably involves some degree of tissue turnover over the year-long sampling period, this strategy was implemented to minimize developmental variability and ensure comparability among sampling points. Floral buds were harvested in December; flowers in February; and fruits in April. The plant material was identified by M. Sc. Gabriel Flores Franco, the curator of HUMO herbarium at the Morelos Autonomous Statal University (Morelos, Mexico), and it was deposited under the voucher number 26349.

### 4.2. Plant Extracts Preparation

The plant material was dried separately (four individuals per collection) in a dark and dry place at room temperature, then ground and sieved to obtain a homogenous powder. After that, extractions were performed using water-methanol mixture of 1:1, *v*/*v* (J. T. Baker, Phillipsburg, NJ, USA) at 1:3 (g/mL) ratio. Each extract was sonicated for 30 min, and filtered with Whatman #2 paper (Marlborough, MA, USA), followed by a second filtration using a 0.45 μm nylon syringe filter (Merck, Boston, MA, USA). The filtered extracts were evaporated under vacuum (Rotavapor R-124, Büchi, Flawil, Switzerland) at 40 °C until dryness.

### 4.3. High-Performance Liquid Chromatography Analysis

The HPLC analysis was carried out to acquire the chromatographic profile of the hydroalcoholic extracts and the quantification of katsumadin in the leaves, floral stages, and fruits from all collected individuals. Chromatographic analyses were performed using a Jasco AS-4150 liquid chromatograph, which included an autosampler, injector, quaternary pump, and an UV-4075 UV/Visible detector (Jasco Corporation, Tokyo, Japan); with a reverse phase column silica C-18, 5 µm, 4.6 mm × 250 mm (Waters, Milford, MA, USA), an isocratic elution composed by acetonitrile-water (15:85), and a flow rate of 1 mL/min performed over 60 min. The solvents used for the chromatographic processes were of HPLC grade (J.T. Baker, Phillipsburg, NJ, USA). Aliquots of 20 μL extracts dissolved in methanol (1 mg/mL) were used as injection volume. ChromNav 2.0 software was used for peak recording. A wavelength of 254 nm was used for peak detections. Chromatographic analyses were performed without a column oven; therefore, all samples were conducted at 25 °C in a room with temperature control. For quantification purposes, a calibration curve was obtained using pure katsumadin to prepare five concentrations within a range of 0.06–1.0 mg/mL, analyzed in triplicate, for a linear response (R^2^ = 0.998) and equation of Y = 3,000,000X + 53,239.

### 4.4. ^1^H Nuclear Magnetic Resonance Analysis

The ^1^H NMR experiments were performed on a JEOL ECZ 600 MHz (JEOL LTD, MA, USA) with a Royal NMR Optimized High Sensitivity 5 mm Probe. Five milligrams of the hydroalcoholic extract were solubilized in 700 μL of CD_3_OD (J.T. Baker, Phillipsburg, NJ, USA). In addition, 450 μL of D_2_O with KH_2_PO_4_ (1.23% p/p) and trimethylsilyl propionic acid (TMSP, 0.01% *w*/*v*) were added to each sample and used as an internal standard for chemical shift referencing and signal normalization. For comparative analysis, the TMSP signal was integrated and normalized to a relative value of 1.0, and the integrals of other resonances were calculated relative to this internal standard. This approach allowed relative comparisons of signal intensities among samples. Given the complexity of plant extracts and the acquisition parameters employed, the ^1^H NMR data were interpreted as semi-quantitative, and comparisons were restricted to relative differences in metabolite signal abundance rather than absolute quantification. All spectra were acquired using the following parameters: spectral resolution of 0.126 Hz/point, pulse width of 4.0 μs (30°), and a relaxation delay (D1) of 2.0 s. The acquired spectra were manually phased and baseline-corrected prior to analysis.

### 4.5. Principal Component Analysis (PCA)

PCA was carried out by integrating the signals of all ^1^H NMR spectra using the Spinworks (v. 4.2.9) software and then analyzed with SIMCA based on the correlation matrix, and the components were obtained by eigenvalue decomposition. Sample materials for PCA analyses were obtained from three independent trees at five different months (December, February, April, June, and August), yielding a total of 15 biological samples (*n* = 3 per month). The DModX plot (distance to the model in X-space) was used to evaluate the goodness of fit for individual samples within the PCA model. PCA models were validated using 7-fold cross-validation and the model quality was assessed using R^2^ and Q^2^ statistics. The methodology was adapted and modified from Cardoso-Taketa et al. [31].

### 4.6. Growth Conditions of Bacterial Strains and Culture

All culture media used for microbiological assays were acquired from Sigma-Aldrich (St. Louis, MO, USA). Reference strains of the Gram-negative bacteria *Salmonella* Typhi (ATCC 6539) and *Pseudomonas aeruginosa* (ATCC 9027) were initially grown in Mueller–Hinton broth and incubated overnight at 37 °C under constant agitation (120 rpm). Long-term preservation of the bacterial stocks was carried out at −85 °C in broth supplemented with 20% glycerol, with routine subculturing performed every 60 days to maintain viability. Unless otherwise stated, all reagents and chemicals employed in this study were of analytical grade and obtained from commercial suppliers. The methodology was adapted from a previously published protocol [5].

### 4.7. Determination of the Minimum Inhibitory and Bactericidal Concentrations

The antibacterial activity of katsumadin was assessed using the microdilution method in a 96-well plate to establish the MIC. Bacterial suspensions were obtained from isolated colonies cultivated in 5 mL of Cation-Adjusted Mueller-Hinton Broth (CAMHB, BDTM, Le Pont de Claix, France) and incubated until the optical density reached 0.08–0.13 at 600 nm, equivalent to a 0.5 McFarland standard. The suspensions were then diluted to achieve a final inoculum of 5 × 10^5^ CFU/mL. After then, 10 µL aliquots of each positive control were plated onto Mueller-Hinton Agar (MH agar) for CFU confirmation.

A stock solution of pure katsumadin was serially diluted two-fold in sterile water to reach final concentrations of 0.5 and 0.25 mg/mL. The assay included several controls: a sterility control (medium without bacteria), positive controls with gentamicin (4 µg/mL), chloramphenicol (8 µg/mL), or ampicillin (8 µg/mL), and a bacterial growth control (medium with inoculum) for each replicate. Following 18 h of incubation at 37 °C, 10 µL of resazurin (22 µM; Merck, Darmstadt, Germany) was added as a cell viability indicator, and plates were incubated for an additional 2 h. The MIC was recorded as the lowest concentration of katsumadin that did not induce a color change to pink.

To determine the MBC, before adding resazurin, aliquots from each well were transferred to MH agar plates (BDTM, Le Pont de Claix, France). The MBC was defined as the lowest concentration at which no bacterial growth on the agar is observed. The antibacterial tests were adapted from the National Committee for Clinical Laboratory Standards (CLSI) guidelines [32].

## 5. Conclusions

This study demonstrates that secondary metabolism in *T. lineata* is significantly influenced by seasonal and developmental factors. Katsumadin showed a marked spatiotemporal accumulation pattern, with higher levels in leaves during the dry season and in early floral stages. The combination of metabolomic profiling and antibacterial assays provides experimental evidence linking seasonal variation with biological activity. These findings expand the phytochemical knowledge of the species and support its traditional medicinal use. Future research should include controlled stress experiments and mechanistic studies to clarify the regulation and ecological function of katsumadin.

## Figures and Tables

**Table 1:** ^1^H NMR spectroscopic data for katsumadin.

	Reported Signals [7] at 500 MHz in CD_3_OD	Leaves	Floral Stages
No.		δ H (mult., *J* in Hz)	
**1**	4.55 (1H, d, *J* = 7.5 Hz)	4.58 (1H, d, *J* = 7.5 Hz)	4.58 (1H, d, *J* = 7.5 Hz)
**2**	3.96 (1H, m)	3.99 (1H, m)	3.99 (1H, m)
**3**	2.5 (1H, dd, *J* = 8.1, 16.1 Hz) 2.84 (1H, dd, *J* = 5.4, 16.1 Hz)	2.53 (1H, dd, *J* = 8.0, 16.1 Hz) 2.87 (1H, dd, *J* = 5.4, 16.1 Hz)	2.53 (1H, dd, *J* = 8.0, 16.1 Hz) 2.87 (1H, dd, *J* = 5.4, 16.1 Hz)
**2′**	6.83 (1H, d, *J* = 1.9 Hz)	6.85 (1H, d, *J* = 1.9 Hz)	6.85 (1H, d, *J* = 1.9 Hz)
**5′**	6.75 (1H, d, *J* = 8.2 Hz)	6.78 (1H, d, *J* = 8.2 Hz)	6.78 (1H, d, *J* = 8.2 Hz)
**6′**	6.72 (1H, dd, *J* = 1.9, 8.2 Hz)	6.75 (1H, dd, *J* = 2.0, 8.2 Hz)	6.75 (1H, dd, *J* = 2.0, 8.2 Hz)
**4″**	5.84 (1H, d, *J* = 2.3 Hz)	5.87 (1H, d, *J* = 2.4 Hz)	5.87 (1H, d, *J* = 2.4 Hz)
**6″**	5.91 (1H, d, *J* = 2.3 Hz)	5.94 (1H, d, *J* = 2.4 Hz)	5.94 (1H, d, *J* = 2.4 Hz)

**Table 2:** MIC and MBC of katsumadin expressed in mg/mL.

Compound and Controls	Bacteria			
	*Salmonella* Typhi		*Pseudomonas aeruginosa*	
	MIC	MBC	MIC	MBC
Katsumadin	0.5	0.5	0.5	0.5
Positive controls				
Gentamicin	0.004	0.004	0.004	0.004
Chloramphenicol	0.008	0.008	-	-
Ampicillin	-	-	0.008	0.008

## Data Availability

The original contributions presented in this study are included in the article/Supplementary Material. Further inquiries can be directed to the corresponding authors.

## Supplementary Materials

The following supporting information can be downloaded at: https://www.mdpi.com/article/10.3390/molecules31060964/s1, Table S1. ^1^H NMR signals from leaf, floral bud, flower, and fruits extracts used to build the five-set and three-set Venn diagrams. Table S2. ^1^H NMR signals from metabolites identified in the leaf, loral bud, flower, and fruit extracts of *T. lineata*. Figure S1. DModX plot for the PCA-X model of leaf extracts from each month. Figure S2. DModX plot for the PCA-X model of the floral bud (FD), flower (FL), and fruit (FR) extracts.

## Abbreviations

The following abbreviations are used in this manuscript:

DModX	Distance to the model in X-space
FD	Floral Bud
FL	Flower
FR	Fruit
HPLC	High-Performance Liquid Chromatography
LC-MS	Liquid Chromatography-Mass Spectrometry
MBC	Minimum bactericidal concentration
MIC	Minimum inhibitory concentration
MH	Mueller-Hinton
NMR	Nuclear Magnetic Resonance
PCA	Principal Component Analysis
