# Supplementary material for: Metabolomic Profiling of Seasonal Katsumadin Production in Ternstroemia lineata
Source: Molecules. 2026 Mar 13;31(6):964. doi: 10.3390/molecules31060964 (PMC13028835; doi:10.3390/molecules31060964)
Supplement: Supplementary file 1 [file molecules-31-00964-s001.zip › molecules-4165770-supplementary.pdf]

Metabolomic Profiling of Seasonal Katsumadin Production in *Ternstroemia lineata*

Alexis Uriel Soto-Díaz[1], María Luisa Villarreal [1], Nahim Salgado Medrano [2], Marcelo Victorio-De los Santos [3], Edda Sciutto [4], José Alejandro Espinosa Cerón [4], Jan Schripsema [5]*, Alexandre Toshirrico Cardoso-Taketa[1]*

Supplementary Materials

	Pg.
**Table S1.** ^{1}H NMR signals from leaf, floral bud, flower, and fruits extracts used to build the five-set and three-set Venn diagrams.	2
**Table S2.** ^{1}H NMR signals from metabolites Identified in the leaf, loral bud, flower, and fruit extracts of *T. lineata*.	24
**Figure S1.** DModX plot for the PCA-X model of leaf extracts from each month.	26
**Figure S2.** DModX plot for the PCA-X model of the floral bud (FD), flower (FL), and fruit (FR) extracts.	26

Table S1. ^{1}H NMR signals from leaf, floral bud, flower, and fruits extracts used to build the five-set and three-set Venn diagrams.

Leaf extract						Floral bud	Flower	Fruit	
**DEC**	**FEB**	**APR**	**JUN**	**AUG**	**Summary**	**DEC**	**FEB**	**APR**	**Summary**
**A**	**B**	**C**	**D**	**E**		**A**	**B**	**C**	
0.856	0.856	0.856	0.856	0.856	ABCDE	0.856	0	0	A
0	0	0	0	0	0	0.882	0.882	0	AB
0	0	0	0	0.876	E	0	0	0.876	C
0.895	0.895	0.895	0.895	0.895	ABCDE	0.895	0	0.895	AC
0	0	0	0.905	0.905	DE	0.905	0.905	0.905	ABC
0	0	0.915	0.915	0	CD	0	0	0	0
0.925	0.925	0.925	0.925	0.925	ABCDE	0.925	0	0	A
0.943	0.943	0.43	0.943	0	ABCD	0.943	0.943	0.943	ABC
0	0	0	0	0	0	0	0.949	0.949	BC
0	0	0	0	0.952	E	0	0	0	0
0.956	0.956	0.956	0.956	0	ABCD	0.956	0.956	0.956	ABC
0	0	0	0	0	0	0	0.96	0.96	BC
0	0	0	0.965	0	D	0	0	0	0
0	0	0	0	0	0	0.977	0.977	0	AB
0	0	0	0	0	0	0	0	0.981	C
0.992	0.992	0.992	0.992	0.992	ABCDE	0.992	0	0	A
0	0	0	0	0	0	0	1.001	0	B
1.009	1.009	1.009	1.009	1.009	ABCDE	1.009	0	0	A
0	0	0	0	0	0	0	1.015	1.015	BC
1.021	1.021	1.021	1.021	1.021	ABCDE	1.021	0	0	A
1.0.31	1.031	1.031	1.031	1.031	ABCDE	1.031	0	0	A

0	0	0	0	0	0	0	1.053	1.053	BC
0	0	0	0	0	0	1.067	0	0	A
1.129	1.129	1.129	1.129	1.129	ABCDE	1.129	0	0	A
1.142	1.142	1.142	1.142	1.142	ABCDE	1.142	0	0	A
1.158	1.158	1.158	0	1.158	ABCE	1.158	1.158	1.158	ABC
0	0	0	0	1.163	E	0	0	0	0
1.171	1.171	1.171	1.171	1.171	ABCDE	1.171	0	0	A
1.181	1.181	1.181	0	1.181	ABCE	1.181	0	0	A
1.189	1.189	1.189	1.189	1.189	ABCDE	1.189	0	0	A
1.208	1.208	1.208	0	1.208	ABCE	1.208	0	0	A
1.222	1.222	1.222	0	1.222	ABCE	1.222	0	0	A
1.23	1.23	1.23	0	1.23	ABCE	1.23	0	0	A
1.242	1.242	1.242	1.242	1.242	ABCDE	1.242	1.242	1.242	ABC
0	0	0	0	0	0	0	0	1.248	C
1.254	1.254	1.254	1.254	0	ABCD	1.254	1.254	0	AB
0	0	0	0	0	0	0	1.262	0	B
1.275	1.275	1.275	0	0	ABC	0	1.275	0	B
1.286	1.286	1.286	0	0	ABC	0	1.286	0	B
1.294	1.294	1.294	1.294	0	ABCD	1.294	1.294	1.294	ABC
1.315	1.315	1.315	1.315	1.315	ABCDE	1.315	1.315	1.315	ABC
1.328	1.328	1.328	1.328	1.328	ABCDE	1.328	1.328	1.328	ABC
0	0	0	1.33	1.33	DE	0	1.33	0	B
0	0	0	0	1.341	E	0	1.34	1.341	BC
0	0	1.345	1.345	1.345	CDE	1.345	1.345	1.345	ABC
0	0	0	0	0	0	0	1.352	1.352	BC
0	0	0	0	0	0	1.361	0	0	A
0	0	0	0	0	0	1.368	0	0	A
0	0	0	0	0	0	1.375	0	0	A

1.39	1.39	1.39	1.39	1.39	ABCDE	1.39	0	0	A
1.479	1.479	1.479	1.479	0	ABCD	1.479	1.479	1.479	ABC
1.494	1.494	1.494	1.494	1.494	ABCDE	1.494	1.494	1.494	ABC
0	0	0	0	1.505	E	1.505	0	0	A
0	0	0	0	0	0	1.516	0	0	A
1.522	1.522	1.522	1.522	1.522	ABCDE	1.522	0	0	A
0	0	0	0	0	0	1.543	0	0	A
1.558	1.558	1.558	1.558	1.558	0	1.558	0	0	A
0	0	0	1.59	0	D	0	0	0	0
0	0	0	1.602	0	D	0	0	0	0
1.64	1.64	1.64	0	0	ABC	0	0	0	0
1.671	1.671	1.671	0	0	ABC	1.671	0	0	A
1.7	1.7	1.7	1.7	1.7	ABCDE	1.7	0	0	A
1.723	1.723	1.723	1.723	1.723	ABCDE	1.723	0	0	A
1.802	1.802	1.802	1.802	1.802	ABCDE	1.802	0	0	A
1.816	1.816	1.816	1.816	1.816	ABCDE	1.816	0	0	A
1.83	1.83	1.83	1.83	1.83	ABCDE	1.83	0	1.83	AC
1.858	1.858	1.858	1.858	1.858	ABCDE	1.858	1.858	1.858	ABC
1.878	1.878	1.878	1.878	1.878	ABCDE	1.878	1.878	1.878	ABC
1.885	1.885	1.885	1.885	1.885	ABCDE	1.885	1.885	1.885	ABC
1.905	1.905	1.905	1.905	1.905	ABCDE	1.905	1.905	1.905	ABC
1.913	1.913	1.913	1.913	1.913	ABCDE	1.913	1.913	1.913	ABC
1.918	1.918	1.918	1.918	1.918	ABCDE	1.918	1.918	1.918	ABC
1.923	1.923	1.923	1.923	1.923	ABCDE	1.923	1.923	1.923	ABC
1.928	1.928	1.928	1.928	1.928	ABCDE	1.928	0	0	A
0	0	0	0	0	0	1.935	1.935	1.935	ABC
0	0	0	0	0	0	1.939	1.939	1.939	ABC
1.943	1.94	1.943	1.943	1.943	ABCDE	1.943	1.943	1.943	ABC

1.947	1.947	1.947	1.947	1.947	ABCDE	0	1.947	1.947	BC
1.952	1.952	1.952	1.952	1.952	ABCDE	0	0	0	0
1.956	1.956	1.956	1.956	1.956	ABCDE	0	0	0	0
1.973	1.973	1.973	1.973	1.973	ABCDE	0	0	0	0
0	0	0	0	0	0	0	1.983	0	B
0	0	0	0	0	0	0	2.001	2.001	BC
0	0	0	0	0	0	2.006	2.006	2.006	ABC
2.012	2.012	2.012	2.012	2.012	ABCDE	2.012	2.012	2.012	ABC
0	0	0	0	0	0	2.015	2.015	2.015	ABC
2.023	2.023	2.023	2.023	2.023	ABCDE	2.023	2.023	2.023	ABC
2.029	2.029	2.029	2.029	2.029	ABCDE	2.029	2.029	2.029	ABC
0	0	0	0	0	0	0	2.033	2.033	BC
2.038	2.038	2.038	2.038	2.038	ABCDE	2.038	2.038	2.038	ABC
2.043	2.043	2.043	2.043	2.043	ABCDE	2.043	2.043	2.043	ABC
0	0	0	0	0	0	0	2.048	2.048	BC
2.052	2.052	2.052	2.052	2.052	ABCDE	2.052	0	0	A
2.058	2.058	2.058	2.058	2.058	ABCDE	2.058	2.058	2.058	ABC
0	0	0	0	0	0	2.125	0	0	A
2.245	2.245	2.245	0	2.245	0	0	0	0	0
2.258	2.258	2.258	0	2.258	0	0	0	0	0
0	0	0	0	0	0	0	2.311	0	B
0	0	0	0	0	0	0	2.367	0	B
0	0	0	0	0	0	2.374	0	2.374	AC
2.399	2.399	2.399	2.399	0	ABCD	2.399	2.399	2.399	ABC
0	0	0	2.425	2.425	DE	0	2.425	2.425	BC
0	0	0	0	2.443	E	2.443	2.443	2.443	ABC
2.458	2.458	2.458	0	0	ABC	2.458	2.458	2.458	ABC
2.472	2.472	2.472	0	0	ABC	2.472	2.472	2.472	ABC

0	0	0	0	0	0	0	2.48	0	B
2.486	2.486	2.486	0	0	ABC	2.486	0	2.486	AC
0	0	0	2.499	0	D	0	0	0	0
0	0	2.504	2.504	0	CD	0	0	0	0
0	0	0	0	0	0	2.51	2.51	2.51	ABC
2.522	2.522	2.522	2.522	0	ABCD	2.522	2.522	2.522	ABC
0	0	2.526	2.526	0	CD	0	2.526	0	B
2.535	2.535	2.535	0	2.535	ABCE	2.535	2.535	0	AB
0	0	0	0	0	0	0	2.541	2.541	BC
2.551	2.551	2.551	2.551	2.551	ABCDE	2.551	2.551	0	AB
0	0	0	0	0	0	2.554	0	2.554	AC
2.567	2.567	2.567	2.567	2.567	ABCDE	0	0	0	0
0	0	0	0	0	0	2.695	2.695	2.695	ABC
0	0	0	0	0	0	2.702	2.702	2.702	ABC
2.707	2.707	2.707	0	0	ABC	2.707	0	2.707	BC
0	0	0	0	0	0	2.729	2.729	2.729	ABC
0	0	0	0	0	0	0	2.733	2.733	BC
2.738	2.738	0	2.738	0	ABD	2.738	0	0	A
2.745	2.745	2.745	2.745	2.745	ABCDE	2.745	0	2.745	AC
2.76	2.76	2.76	2.76	2.76	ABCDE	0	0	0	0
2.77	2.77	2.77	2.77	0	ABCD	0	0	0	0
2.777	2.777	2.777	2.777	2.777	ABCDE	2.777	0	0	A
0	0	2.788	0	0	C	0	0	0	0
2.796	2.796	2.796	2.796	2.796	ABCDE	2.796	2.796	2.796	ABC
0	0	0	0	0	0	0	0	2.799	C
2.81	2.81	2.81	2.81	2.81	ABCDE	2.81	2.81	2.81	ABC
0	0	0	0	0	0	0	2.818	2.88	BC
2.824	2.824	2.824	2.824	2.824	ABCDE	2.824	2.824	2.824	ABC

0	0	0	2.835	2.835	DE	0	0	0	0
0	0	0	0	0	0	0	2.842	2.842	BC
0	0	0	0	0	0	0	2.846	0	B
2.853	2.853	2.853	2.853	2.853	ABCDE	2.853	0	2.853	AC
0	0	0	0	0	0	0	2.855	2.855	BC
0	0	0	0	0	0	0	2.859	2.859	BC
0	0	0	0	2.865	E	0	0	0	0
0	0	0	0	2.87	E	0	0	2.87	C
0	0	0	0	0	0	2.875	2.875	0	AB
2.882	2.882	2.882	2.882	2.882	ABCDE	0	0	0	0
0	0	0	2.889	0	D	0	0	0	0
2.892	2.892	2.892	2.892	2.892	ABCDE	0	0	0	0
2.90	2.90	2.90	2.90	0	ABCD	0	0	0	0
2.91	2.91	0	0	0	AB	0	0	0	0
2.921	2.921	2.921	2.921	0	ABCD	0	0	0	0
2.934	2.934	2.934	0	0	ABC	2.934	2.934	2.934	ABC
0	0	0	0	0	0	0	2.94	2.94	BC
2.948	2.948	2.948	2.948	0	ABCD	0	0	0	0
2.964	2.964	2.964	0	2.964	ABCE	0	0	2.964	C
0	0	0	0	0	0	0	0	2.974	C
3.101	3.101	0	0	3.101	ABE	3.101	0	0	A
3.126	3.126	3.126	3.126	3.126	ABCDE	3.126	0	0	A
3.146	3.146	3.146	3.146	3.146	ABCDE	3.146	0	0	A
0	0	3.152	0	0	C	0	0	0	0
3.158	3.158	3.158	3.158	3.158	ABCDE	3.158	0	0	A
0	0	0	3.167	3.167	DE	0	0	0	0
0	0	0	3.17	3.17	DE	0	0	0	0
0	0	0	3.174	3.174	DE	0	0	3.174	C

0	0	0	0	0	0	0	3.178	3.178	BC
3.184	3.184	3.184	3.184	3.184	ABCDE	3.184	3.184	3.184	ABC
0	0	0	3.195	3.195	DE	0	0	0	0
0	3.199	0	3.199	0	BD	0	3.199	3.199	BC
0	3.201	0	0	0	B	3.201	3.201	3.201	ABC
3.203	3.203	3.203	3.203	3.203	ABCDE	3.203	0	3.203	AC
0	0	0	0	0	0	0	3.208	0	B
0	0	0	3.214	3.214	DE	0	0	0	0
3.218	3.218	3.218	3.218	3.218	ABCDE	3.218	3.218	3.218	ABC
0	0	0	0	0	0	0	3.221	3.221	BC
0	3.226	0	0	3.226	BE	0	3.226	3.226	BC
0	3.228	0	0	3.228	BE	0	3.228	3.228	BC
3.23	3.23	3.23	3.23	3.23	ABCDE	3.23	0	0	A
0	0	0	0	0	0	3.237	0	3.237	AC
0	0	0	0	0	0	0	3.239	0	B
3.241	3.241	3.241	3.241	3.241	ABCDE	3.241	0	0	A
0	3.242	3.242	0	0	BC	0	3.242	3.242	BC
0	0	0	0	0	0	3.253	3.253	3.253	ABC
3.26	3.26	3.26	3.26	3.26	ABCDE	3.261	3.261	3.261	ABC
3.27	3.27	3.27	3.27	3.27	ABCDE	3.27	0	3.27	AC
0	0	0	0	0	0	0	0	3.274	C
0	0	0	0	0	0	0	0	3.278	C
3.298	3.298	3.298	3.298	3.298	ABCDE	3.298	0	0	A
3.336	3.336	3.336	3.336	3.336	ABCDE	0	3.336	3.336	BC
0	0	0	0	0	0	0	3.34	3.34	BC
3.343	3.343	3.343	3.343	3.343	ABCDE	3.343	3.343	3.343	ABC
0	0	0	0	0	0	0	3.348	3.348	BC
3.354	3.354	3.354	3.354	3.354	ABCDE	3.354	3.354	3.354	ABC

0	0	0	0	0	0	0	0	3.358	C
3.361	3.361	3.361	3.361	3.361	ABCDE	3.361	3.361	3.361	ABC
3.368	3.368	3.368	3.368	3.368	ABCDE	3.368	3.368	3.368	ABC
3.379	3.379	3.379	3.379	3.379	ABCDE	3.379	3.379	3.379	ABC
3.384	3.384	3.384	3.384	3.384	ABCDE	3.384	3.384	3.384	ABC
0	0	0	0	0	0	0	3.388	3.388	BC
3.391	3.391	3.391	3.91	3.391	ABCDE	3.391	0	0	A
3.398	3.398	3.398	3.398	3.398	ABCDE	3.398	3.398	3.398	ABC
3.402	3.402	3.402	3.402	3.402	ABCDE	3.402	3.402	3.402	ABC
0	0	0	3.404	3.404	DE	0	3.404	3.404	BC
0	0	0	0	0	0	0	3.407	3.407	BC
0	0	0	3.413	3.413	DE	3.413	0	0	A
0	0	0	3.416	3.416	DE	0	3.416	3.416	BC
3.418	3.418	3.418	3.418	3.418	ABCDE	0	0	0	0
0	0	0	0	0	0	3.423	3.423	3.423	ABC
0	0	0	0	0	0	3.426	0	0	A
0	0	0	3.433	3.433	DE	0	0	0	0
3.438	3.438	3.438	3.438	3.438	ABCDE	3.438	3.438	3.438	ABC
0	0	0	0	0	0	0	3.441	3.441	BC
0	0	3.447	0	0	C	3.447	0	0	A
0	0	0	3.453	3.453	DE	0	3.453	3.453	BC
3.455	3.455	3.455	3.455	3.455	ABCDE	3.455	3.455	0	AB
0	0	0	0	0	0	3.46	3.46	3.46	ABC
0	0	0	3.464	0	D	0	3.464	3.464	BC
3.466	3.466	3.466	3.466	3.466	ABCDE	3.466	3.466	0	AB
0	0	0	3.473	3.473	DE	0	0	0	0
3.477	3.477	3.477	3.477	3.477	ABCDE	3.477	3.477	3.477	ABC
0	0	0	3.48	3.48	DE	0	3.48	3.48	BC

0	0	0	0	0	0	3.483	3.48	3.483	ABC
3.487	3.487	3.487	3.487	3.487	ABCDE	3.487	3.487	3.487	ABC
0	0	0	3.493	0	D	0	0	0	0
3.498	3.498	3.498	3.498	3.498	ABCD	3.498	3.498	3.498	ABC
3.51	3.51	3.51	3.51	3.51	ABCDE	3.51	3.51	3.51	ABC
0	0	0	3.513	0	D	0	3.513	3.513	BC
3.518	3.518	3.518	3.518	3.518	ABCDE	3.518	0	0	A
0	0	0	0	0	0	0	0	3.521	C
0	0	0	0	0	0	3.523	3.523	3.523	ABC
3.526	3.526	3.526	3.526	3.526	ABCDE	3.526	0	0	A
3.534	3.534	3.534	3.534	3.534	ABCDE	3.534	3.534	3.534	ABC
3.549	3.549	3.549	3.549	3.549	ABCDE	3.549	3.549	3.549	ABC
0	0	0	0	0	0	0	3.551	3.551	BC
0	0	0	0	0	0	0	0	3.557	C
3.565	3.565	3.565	3.565	3.565	ABCDE	3.565	3.565	3.565	ABC
0	0	0	0	0	0	3.573	3.573	3.573	ABC
3.577	3.577	3.577	3.577	0	ABCD	3.577	0	0	A
0	3.586	3.586	3.586	3.586	BCDE	0	0	0	0
0	0	0	0	0	0	0	3.589	3.589	BC
0	0	0	0	0	0	3.591	0	0	A
3.599	3.599	3.599	3.599	3.599	ABCDE	3.599	3.599	3.599	ABC
0	0	0	0	0	0	0	3.607	3.607	BC
0	0	0	0	0	0	0	0	3.611	C
3.614	3.614	3.614	3.614	3.614	ABCDE	3.614	3.614	3.614	ABC
0	0	0	0	3.616	E	0	0	0	0
3.621	3.621	3.621	3.621	3.621	ABCDE	3.621	3.621	3.621	ABC
0	0	0	3.625	0	D	0	0	3.625	C
0	0	0	3.632	3.632	DE	0	3.632	3.632	BC

0	0	0	3.637	3.637	DE	3.637	0	0	A
0	0	0	0	0	0	3.641	3.641	3.641	ABC
0	0	0	0	3.643	E	0	0	3.643	C
0	0	0	3.647	3.647	DE	0	3.647	3.647	BC
0	0	0	3.651	3.651	DE	0	0	0	0
3.655	3.655	3.655	3.655	3.655	ABCDE	3.655	3.655	3.655	ABC
0	0	0	0	0	0	0	3.659	0	B
0	0	0	0	3.663	E	0	0	0	0
3.668	3.668	3.668	3.668	3.668	ABCDE	3.668	3.668	3.668	ABC
0	0	0	0	0	0	3.674	3.674	3.674	ABC
3.68	3.68	3.68	3.68	3.68	ABCDE	3.68	3.68	3.68	ABC
3.692	3.692	3.692	3.692	3.692	ABCDE	3.692	3.69	3.692	ABC
3.698	3.698	3.698	3.698	3.698	ABCDE	3.698	3.698	3.698	ABC
3.705	3.705	3.705	3.705	3.705	ABCDE	3.705	3.705	3.705	ABC
3.715	3.715	3.715	3.715	3.715	ABCDE	3.715	3.715	3.715	ABC
0	0	0	0	0	0	0	3.722	0	B
3.724	3.724	3.724	3.724	3.724	ABCDE	3.724	3.724	3.724	ABC
0	0	0	3.732	3.732	DE	3.732	3.732	3.732	ABC
3.743	3.743	3.743	3.743	3.743	ABCDE	3.743	3.743	3.743	ABC
0	3.757	3.757	3.757	3.757	BCDE	0	0	0	0
0	3.762	3.762	3.762	3.762	BCDE	0	3.762	3.762	BC
3.764	3.764	3.764	3.764	3.764	ABCDE	3.764	0	0	A
0	0	0	0	0	0	3.768	3.768	3.768	ABC
3.784	3.784	3.784	3.784	3.784	ABCDE	3.784	3.784	3.784	ABC
0	0	3.787	3.787	3.787	CDE	0	3.787	3.787	BC
3.792	3.792	3.792	3.792	3.792	ABCDE	3.792	3.792	3.792	ABC
3.797	3.797	3.797	3.797	3.797	ABCDE	3.797	3.797	3.797	ABC
3.803	3.803	3.803	3.803	3.803	ABCDE	3.803	3.803	3.803	ABC

3.809	3.809	3.809	3.809	0	ABCD	3.809	3.809	3.809	ABC
0	0	0	0	0	0	0	0	3.815	C
0	3.82	0	0	0	B	0	0	0	0
3.823	3.823	3.823	3.823	3.823	ABCDE	3.823	3.823	3.823	ABC
0	3.828	0	0	0	B	0	0	3.828	C
3.83	3.83	3.83	3.83	3.83	ABCDE	3.83	3.83	3.83	ABC
3.836	3.836	3.836	3.836	3.836	ABCDE	3.836	3.836	3.836	ABC
0	3.842	3.842	3.842	0	BCD	0	0	0	0
3.844	3.844	3.844	3.844	3.844	ABCDE	3.844	0	0	A
0	0	3.847	0	0	C	0	3.847	3.847	BC
3.854	3.854	3.854	3.854	3.854	ABCDE	3.854	3.854	3.854	ABC
0	0	0	3.86	3.86	DE	0	0	0	0
3.864	0	0	3.864	3.864	ADE	3.864	3.864	3.864	0
3.867	3.867	3.867	3.867	3.867	ABCDE	3.867	3.867	3.867	ABC
0	0	0	0	0	0	0	3.869	0	B
0	0	0	0	0	0	0	3.873	3.873	BC
0	0	0	0	0	0	0	3.878	3.878	BC
0	0	0	3.884	3.884	DE	0	0	0	0
0	0	0	0	0	0	3.889	3.889	3.889	ABC
3.892	3.892	3.892	3.892	3.892	ABCDE	3.892	3.892	3.892	ABC
0	0	0	0	0	0	0	3.908	3.908	BC
0	0	0	0	0	0	3.912	0	0	A
3.925	0	3.925	3.925	3.925	ACDE	3.925	3.925	0	AB
0	0	0	3.933	3.933	DE	0	0	3.933	C
0	0	0	3.936	3.936	DE	0	3.936	0	B
0	0	0	3.94	3.94	DE	0	3.94	3.94	BC
0	0	0	3.943	3.943	DE	3.943	3.943	3.943	ABC
0	0	0	3.947	3.947	DE	3.947	3.947	3.947	ABC

0	0	0	0	0	0	3.95	3.95	3.95	ABC
0	0	0	0	0	0	0	3.959	3.959	BC
3.964	3.964	3.964	3.964	3.964	ABCDE	3.964	3.964	3.964	ABC
3.973	3.973	3.973	3.973	3.973	ABCDE	3.973	3.973	3.973	ABC
0	0	0	0	0	0	0	3.976	3.976	BC
0	3.979	3.979	3.979	3.979	BCDE	3.979	3.979	3.979	ABC
3.983	3.983	3.983	3.983	3.983	ABCDE	3.983	3.983	3.983	ABC
3.991	3.991	3.991	3.991	3.991	ABCDE	3.991	3.991	3.991	ABC
0	3.995	3.995	3.995	3.995	BCDE	0	3.995	3.995	BC
4	4	4	4	4	ABCDE	4	0	0	A
0	0	0	4.08	4.08	DE	0	4.08	4.08	BC
0	0	0	4.01	4.01	DE	0	4.01	4.01	BC
4.014	4.014	4.014	4.014	4.014	ABCDE	4.014	4.014	0	BC
0	0	0	0	4.016	E	0	0	0	0
0	0	0	4.022	4.022	DE	4.022	0	0	A
0	0	0	4.027	4.027	DE	0	0	0	0
4.031	4.031	4.031	4.031	4.031	ABCDE	4.031	4.031	4.031	ABC
0	0	0	4.036	4.036	DE	4.036	4.036	4.036	ABC
0	0	0	0	0	0	0	4.04	4.04	BC
4.047	4.047	4.047	4.047	4.047	ABCDE	4.047	4.047	4.047	ABC
4.057	4.057	4.057	4.057	4.057	ABCDE	4.057	0	4.057	AC
0	4.059	4.059	4.059	4.059	BCDE	0	0	0	0
4.067	4.067	4.067	0	4.067	ABCE	4.067	4.067	4.067	ABC
4.078	4.078	4.078	4.078	4.078	ABCDE	4.078	4.078	4.078	ABC
4.084	4.084	4.084	4.084	4.084	ABCDE	4.084	4.084	4.084	ABC
0	4.086	4.086	4.086	4.086	BCDE	4.086	4.086	4.086	ABC
0	0	0	0	0	0	0	4.091	4.091	BC
4.097	4.097	4.097	4.097	4.097	ABCDE	4.097	4.097	4.097	ABC

0	4.102	0	4.102	0	BD	0	4.102	4.102	BC
4.105	4.105	4.105	4.105	4.105	ABCDE	4.105	4.105	4.105	ABC
0	4.108	0	4.108	0	BD	0	4.108	4.108	BC
4.113	4.113	4.113	4.113	4.113	ABCDE	4.113	4.113	4.113	ABC
4.119	4.119	4.119	4.119	4.119	ABCDE	4.119	0	4.119	AC
0	0	0	0	0	0	4.127	0	4.127	AC
0	0	0	0	0	0	0	0	4.134	C
4.16	4.16	4.16	4.16	4.16	ABCDE	4.16	4.16	4.16	ABC
4.18	4.18	4.18	4.18	4.18	ABCDE	4.18	4.18	4.18	ABC
0	0	0	0	0	0	4.19	0	0	A
4.23	4.23	4.23	4.23	4.23	ABCDE	4.23	0	0	A
4.24	4.24	4.24	4.24	4.24	ABCDE	4.24	0	0	A
4.25	4.25	4.25	4.25	4.25	ABCDE	4.25	0	0	A
4.26	4.26	4.26	4.26	0	ABCD	4.26	0	0	A
4.274	4.274	4.274	4.274	4.274	ABCDE	4.274	0	0	A
0	0	0	0	0	0	0	4.277	4.277	BC
0	0	0	0	4.283	E	0	4.283	0	B
4.28	4.28	4.28	4.28	0	ABCD	4.28	0	4.28	AC
4.294	4.294	0	4.294	0	ABD	4.294	4.294	4.29	ABC
0	4.301	0	0	4.301	BE	0	4.301	0	B
4.305	4.305	4.305	4.305	4.305	ABCDE	4.305	0	4.304	AC
4.31	4.31	4.31	4.31	4.31	ABCDE	4.31	0	4.31	AC
0	4.317	0	0	0	B	0	0	4.317	C
4.319	4.319	4.319	0	0	ABC	4.319	0	0	A
4.331	4.331	4.331	4.331	4.331	ABCDE	0	0	0	0
4.349	4.349	4.349	4.349	4.349	ABCDE	0	0	0	0
4.366	4.366	4.366	4.366	4.366	ABCDE	4.366	0	4.366	AC
0	0	0	0	0	0	0	0	4.383	C

4.387	4.387	4.387	4.387	4.387	ABCDE	4.387	4.387	4.387	ABC
4.402	4.402	4.402	4.402	4.402	ABCDE	4.402	4.402	4.402	ABC
0	0	0	4.415	4.415	DE	4.415	4.415	4.415	ABC
4.42	4.42	4.42	4.42	4.42	ABCDE	4.42	4.42	4.42	ABC
0	0	0	0	4.438	E	4.438	0	0	A
4.441	4.441	4.441	4.441	4.441	ABCDE	4.441	4.441	4.441	ABC
4.446	4.446	4.446	4.446	4.446	ABCDE	4.446	0	4.446	AC
4.452	4.452	4.452	4.452	4.452	ABCDE	4.452	0	0	A
0	4.455	0	0	0	B	0	4.45	0	B
0	4.464	4.464	0	0	BC	0	0	0	0
0	4.468	4.468	0	0	BC	0	4.468	4.468	BC
4.472	4.472	4.472	4.472	4.472	ABCDE	4.472	4.472	4.472	ABC
0	0	0	0	0	0	4.479	0	0	A
0	0	0	0	0	0	4.486	4.486	4.486	ABC
4.489	4.489	4.489	4.489	4.489	ABCDE	4.489	4.489	4.489	ABC
4.507	4.507	4.507	4.507	4.507	ABCDE	0	0	0	0
4.523	4.5230	4.523	4.523	4.523	ABCDE	4.523	4.523	0	AB
0	0	4.54	0	4.54	CE	4.54	4.54	0	AB
0	0	0	0	0	0	0	4.551	0	B
4.579	4.579	4.579	4.579	4.579	ABCDE	4.579	4.579	4.579	ABC
4.593	4.593	4.593	4.593	4.593	ABCDE	4.593	5.593	4.593	ABC
4.6	4.6	4.6	0	4.6	ABCE	0	0	0	0
0	4.619	4.619	0	0	BC	0	0	0	0
4.621	4.621	4.621	4.621	0	ABCD	4.621	0	0	A
0	4.627	4.627	0	0	BC	0	0	0	0
0	4.632	4.632	4.632	0	BCD	0	0	0	0
4.638	4.638	4.638	4.638	4.638	ABCDE	4.638	0	0	A
0	4.643	4.643	0	4.643	BCE	0	0	0	0

0	4.646	4.646	0	4.646	BCE	4.646	0	0	A
4.651	4.651	4.651	0	4.651	ABCE	0	0	0	0
4.662	4.662	4.662	0	0	ABC	4.662	0	0	A
4.668	4.668	4.668	4.668	0	ABCD	4.668	0	0	A
0	4.671	0	0	0	B	4.671	0	0	A
0	0	4.675	0	0	C	0	0	0	0
0	0	0	0	0	0	4.688	4.688	4.688	ABC
0	0	0	0	0	0	4.703	7.703	4.703	ABC
0	0	4.917	0	0	C	0	0	0	0
4.942	4.942	4.942	4.942	4.942	ABCDE	4.942	0	0	A
4.961	4.961	4.961	4.961	4.961	ABCDE	4.961	0	0	A
4.98	4.98	4.98	4.98	4.98	ABCDE	4.98	0	0	A
5.092	0	0	0	5.092	AE	0	0	0	0
0	0	0	0	5.101	E	0	0	0	0
0	0	0	0	5.107	E	0	0	0	0
5.15	5.15	5.15	5.15	5.15	ABCDE	5.15	0	5.15	AC
5.16	5.16	5.16	5.16	5.16	ABCDE	5.16	0	0	A
5.18	5.18	5.18	5.18	5.18	ABCDE	5.18	5.18	5.18	ABC
5.19	5.19	5.19	5.19	5.19	ABCDE	5.19	5.19	5.19	ABC
5.4	5.4	5.4	5.4	5.4	ABCDE	5.4	5.4	5.4	ABC
5.41	5.41	5.41	5.41	5.41	ABCDE	5.41	5.41	5.41	ABC
0	0	0	0	0	0	5.423	0	0	A
0	0	0	0	0	0	5.764	0	0	A
0	0	0	0	0	0	5.768	0	0	A
0	0	0	0	0	0	0	5.949	5.949	BC
0	0	0	0	0	0	5.954	5.954	5.954	ABC
0	0	0	0	0	0	5.958	0	0	A
0	0	0	0	0	0	5.966	5.966	0	AC

0	0	0	0	0	0	5.971	5.966	0	AC
0	0	0	0	0	0	0	6.035	6.035	BC
0	0	0	0	0	0	0	6.036	0	B
0	0	0	0	0	0	6.039	6.039	6.039	ABC
0	0	0	0	0	0	6.403	0	0	A
0	0	0	0	0	0	6.122	6.122	6.122	ABC
0	0	0	6.166	0	D	0	0	0	0
0	0	6.17	0	0	C	0	0	0	0
0	6.172	6.172	6.172	0	BCD	6.172	6.172	0	AB
6.177	6.177	6.177	6.177	0	ABCD	6.177	6.176	0	AB
0	0	0	6.183	0	D	6.183	6.183	0	AB
0	0	0	0	0	0	6.186	6.186	0	AB
0	0	0	0	0	0	0	6.188	0	B
6.192	6.192	6.192	6.192	6.192	ABCDE	6.192	6.192	6.192	ABC
0	0	0	6.196	0	D	0	6.196	6.196	BC
6.198	6.198	6.198	6.198	6.198	ABCDE	6.198	0	0	A
0	0	0	0	0	0	6.204	6.204	6.204	ABC
0	0	0	0	0	0	0	6.208	0	B
6.211	6.211	6.211	6.211	0	ABCD	6.211	6.211	0	AB
0	0	0	6.216	0	D	0	6.216	0	B
0	0	0	6.220	0	D	0	6.221	6.221	BC
0	0	0	0	0	0	6.225	6.225	0	AB
0	0	0	0	0	0	6.23	6.23	6.23	ABC
0	0	0	0	0	0	0	6.233	0	B
0	0	0	0	0	0	0	6.235	0	B
0	0	0	0	0	0	0	6.238	0	B
0	0	0	0	0	0	6.246	6.246	0	AB
0	0	0	0	0	0	0	6.249	0	B

0	0	0	0	0	0	6.26	0	0	A
6.268	6.268	6.268	6.268	0	ABCD	6.268	0	0	A
6.271	6.271	6.271	6.271	0	ABCD	6.271	0	0	A
0	0	0	0	0	0	6.279	0	0	A
0	6.308	6.308	6.308	6.308	BCDE	6.308	0	0	A
0	6.312	6.312	6.312	6.312	BCDE	6.312	0	0	A
0	6.315	0	6.315	0	BD	0	0	0	0
6.321	6.321	6.321	6.321	0	ABCD	0	0	0	0
6.436	6.436	6.436	6.436	0	ABCD	0	0	0	0
6.44	6.44	6.44	6.44	0	ABCD	0	0	0	0
6.505	6.505	6.505	6.505	0	ABCD	0	0	0	0
0	0	6.515	6.515	0	CD	0	0	0	0
6.522	6.522	6.522	6.522	6.522	ABCDE	6.522	0	0	A
6.526	6.526	6.526	6.526	6.526	ABCDE	6.526	0	6.526	AC
0	0	6.532	0	0	C	6.532	0	6.532	AC
6.537	6.537	6.537	6.537	6.537	ABCDE	0	0	0	0
6.54	6.54	6.54	6.54	6.54	ABCDE	6.54	6.54	6.54	ABC
0	0	6.553	6.553	0	CD	0	0	0	0
6.556	6.556	6.556	6.556	0	ABCD	6.556	0	0	A
0	0	6.562	6.562	0	CD	6.562	6.562	6.562	ABC
0	0	0	0	0	0	0	6.566	6.56	BC
6.592	6.59	6.592	6.592	0	ABCD	6.592	0	0	A
6.594	6.594	6.594	6.594	0	ABCD	6.594	6.594	0	AB
6.608	6.608	6.608	6.608	6.608	ABCDE	6.608	6.608	0	AB
6.612	6.612	6.612	6.612	6.612	ABCDE	6.612	6.612	0	AB
0	0	0	0	0	0	0	6.614	6.614	BC
0	6.624	6.624	6.624	6.624	BCDE	0	6.624	6.624	BC
6.629	6.629	6.629	6.629	6.629	ABCDE	6.629	6.629	6.629	ABC

0	0	0	0	0	0	6.634	6.634	6.634	ABC
0	0	6.64	6.64	6.64	CDE	0	0	6.64	C
6.644	6.644	6.644	6.644	6.644	ABCDE	6.644	0	6.644	AC
0	6.648	6.648	6.648	6.648	BCDE	0	0	0	0
0	0	0	0	0	0	0	6.653	6.653	BC
0	0	0	0	0	0	6.658	6.658	0	AB
0	6.662	0	6.662	6.662	BDE	0	0	0	0
6.66	6.66	6.66	6.66	6.66	ABCDE	0	6.66	6.66	BC
0	6.675	6.675	6.675	0	BCD	6.675	0	6.675	AC
0	0	0	0	0	0	6.677	0	6.67	AC
6.68	6.68	6.68	6.68	6.68	ABCDE	6.68	6.68	6.68	ABC
6.69	6.69	6.69	6.69	0	ABCD	6.69	6.69	6.69	ABC
0	0	0	0	0	0	6.694	6.694	6.694	ABC
0	0	0	6.702	0	D	6.702	0	0	A
0	6.705	6.705	6.705	0	BCD	6.705	0	6.705	AC
6.708	6.708	6.708	6.708	6.708	ABCDE	0	0	0	0
0	0	0	0	0	0	6.712	6.712	6.712	ABC
0	0	0	0	0	0	6.718	6.718	0	AB
6.723	6.723	6.723	6.723	6.723	ABCDE	0	0	0	0
0	0	0	0	0	0	6.727	6.727	6.727	ABC
6.735	6.735	6.735	6.735	6.735	ABCDE	0	0	0	0
0	0	0	6.714	6.741	DE	0	0	6.741	C
6.745	6.745	6.745	6.745	6.745	ABCDE	0	6.745	0	B
0	6.748	0	0	6.748	BE	6.748	0	0	A
6.752	6.752	6.752	6.752	0	ABCD	0	6.752	6.752	BC
0	0	0	0	6.756	E	6.756	0	0	A
6.759	6.759	6.759	6.759	0	ABCD	0	0	0	0
0	0	0	6.764	6.764	DE	6.764	6.764	0	AB

0	6.76	0	6.76	6.76	BDE	0	6.76	6.76	BC
6.771	6.771	6.771	6.771	6.771	ABCDE	6.771	6.771	6.771	ABC
0	6.775	6.775	6.775	6.775	BCDE	6.775	0	6.775	AC
0	6.779	6.779	6.779	6.779	BCDE	6.779	6.779	6.779	ABC
0	0	0	0	0	0	6.78	6.78	6.78	ABC
0	6.784	6.784	6.784	6.784	BCDE	0	6.785	6.785	BC
6.787	6.787	6.787	6.787	6.787	ABCDE	6.787	6.787	0	AB
0	0	0	0	0	0	6.792	6.792	6.792	ABC
6.795	6.795	6.795	6.795	6.795	ABCDE	0	6.795	6.795	BC
0	0	0	0	0	0	6.798	6.798	6.798	ABC
0	0	0	6.8	6.8	DE	6.8	6.8	6.8	ABC
6.805	6.805	6.805	6.805	6.805	ABCDE	6.805	6.805	0	AB
6.808	6.808	6.808	6.808	6.808	ABCDE	0	6.808	0	B
0	0	0	0	0	0	6.812	6.812	6.812	BAC
0	0	0	0	0	0	6.817	0	0	A
6.82	6.82	6.82	6.82	6.82	ABCDE	0	6.82	6.82	BC
0	0	0	0	0	0	6.825	6.825	0	AB
0	0	0	0	0	0	0	0	6.839	C
0	0	0	0	0	0	0	6.848	0	B
0	6.86	6.86	0	0	BC	6.86	6.86	6.86	ABC
0	0	0	0	0	0	6.866	6.866	6.86	ABC
0	0	0	0	0	0	6.89	6.89	6.89	abc
6.899	6.899	6.899	6.899	6.899	ABCDE	0	0	0	0
0	0	0	6.91	0	D	0	0	0	0
0	0	0	6.914	0	D	0	0	0	0
0	0	0	0	6.925	E	0	0	0	0
0	0	0	0	6.928	E	0	0	0	0
0	0	0	0	0	0	6.938	6.938	6.938	ABC

0	0	0	0	0	0	6.942	6.942	6.942	ABC
0	0	0	0	0	0	0	6.949	0	B
0	0	0	0	0	0	0	6.955	6.955	BC
0	0	0	0	0	0	0	6.959	0	B
6.967	6.967	6.976	6.967	0	ABCD	0	6.967	6.967	BC
0	0	0	0	0	0	0	6.972	6.972	BC
6.98	6.98	6.98	6.98	6.98	ABCDE	0	6.98	6.98	BC
6.986	6.986	6.986	6.986	6.986	ABCDE	6.986	6.986	0	AB
0	0	0	0	0	0	0	6.988	0	B
6.997	6.997	6.997	6.997	6.997	ABCDE	0	6.997	6.997	BC
0	7	7	7	0	BCD	0	0	7	C
7.003	7.003	7.033	7.003	7.003	ABCDE	7.003	7.003	0	AB
7.009	7.009	0	7.009	7.009	ABDE	0	7.009	7.009	BC
7.02	7.02	7.02	7.02	7.02	ABCDE	0	0	0	0
7.026	7.026	7.026	7.026	7.026	ABCDE	0	0	0	0
0	0	0	0	0	0	7.031	7.031	0	AB
0	0	0	0	0	0	7.036	7.036	7.036	ABC
0	0	0	0	0	0	7.047	7.047	0	AB
7.051	7.051	7.051	7.051	7.051	ABCDE	7.051	0	7.051	AC
0	7.055	7.055	7.055	0	BCD	0	7.055	0	B
0	0	0	0	0	0	7.059	7.059	0	AB
0	7.063	7.063	7.063	7.063	BCDE	0	0	0	0
7.069	7.069	7.069	7.069	7.069	ABCDE	7.069	7.069	7.069	ABC
0	7.075	7.075	7.075	0	BCD	0	7.075	0	B
7.087	7.087	7.087	7.087	7.087	ABCDE	0	0	0	0
0	0	0	7.091	0	D	7.091	7.091	7.091	ABC
0	0	0	7.096	0	D	7.096	7.096	7.096	ABC
0	0	0	0	0	0	0	7.099	7.099	BC

7.102	7.102	7.102	7.102	7.102	ABCDE	7.102	7.102	0	AB
0	0	0	7.107	0	D	0	0	0	0
0	0	0	7.11	0	D	7.11	7.11	7.11	ABC
0	0	0	7.115	7.115	DE	7.115	7.115	7.155	ABC
7.118	7.118	7.118	7.118	7.118	ABCDE	7.118	7.118	7.118	ABC
0	0	0	7.126	0	D	7.126	7.126	0	AB
0	0	0	0	0	0	7.13	7.13	0	AB
0	0	0	7.135	0	D	0	7.135	7.135	BC
0	0	0	0	0	0	7.137	0	0	A
0	0	0	7.142	0	D	0	0	0	0
0	0	0	7.146	0	D	0	7.146	0	B
0	0	0	7.15	0	D	7.15	7.15	0	AB
0	0	0	0	0	0	0	7.155	0	B
7.163	7.163	7.163	7.163	7.163	ABCDE	0	0	0	0
0	0	0	0	0	0	7.174	7.174	0	AB
7.18	7.18	7.18	7.18	7.18	ABCDE	0	0	0	0
0	0	0	7.183	0	D	0	0	0	0
0	0	0	0	0	0	0	7.312	0	B
0	0	0	0	0	0	0	7.329	0	B
0	0	0	7.365	0	D	0	0	0	0
0	0	0	7.37	0	D	0	7.37	0	B
0	7.4	7.4	0	0	BC	0	7.4	0	B
0	0	7.41	0	0	C	0	0	0	0
0	7.43	7.43	7.43	0	BCD	0	0	0	0
0	0	0	0	0	0	7.827	0	0	A
0	0	0	0	0	0	7.831	0	0	A
0	0	0	8.082	8.082	DE	0	0	0	0
0	0	0	8.1	8.1	DE	0	0	0	0

0	0	8.416	8.416	0	CD	0	0	0	0
0	0	8.151	8.151	0	CD	0	0	0	0
8.462	8.462	8.462	8.462	8.462	ABCDE	8.462	8.462	0	AB

Table S2. ^{1}H NMR signals from metabolites Identified in the leaf, loral bud, flower, and fruit extracts of *T. lineata*.

**Identified compounds in the leaf extracts from *T. lineata* according to their ^{1}H NMR signals**	
**Compound**	**Identified signal**
Sucrose	δ 5.41 (d, *J*= 3.97 Hz)
	δ 4.18 (d, *J*= 8.5 Hz)
	δ 3.67 (s)
	δ 3.54 (dd, *J*= 9.46 Hz)
	δ 3.54 (dd, *J*= 3.66 Hz)
	δ 4.04 (dd, *J*= 8.24 Hz)
α-Glucose	δ 5.19 (d, *J*= 3.66 Hz)
β -Glucose	δ 4.58 (d, *J*= 7.63 Hz)
Fructose	δ 3.89 (d, *J*= 2.8 Hz)
	δ 3.53 (d, *J*= 11.7 Hz)
Threonine	δ 3.58 (d, *J*= 6.41Hz)
	δ 4.24 (m)
Alanine	δ 1.48 (d, *J*= 7.32 Hz)
	δ 3.8 (m)
Valine	δ 0.95 (d, *J*= 6.71 Hz)
	δ 1.02 (d, *J*= 6.1 Hz)
	δ 3.62 (d, *J*= 4.27 Hz)
Tyrosine	δ 7.18 (t, *J*= 8.5 Hz)
Adenine	δ 8.08 (s)

	δ 8.1 (s)
**Identified compounds in the floral bud, flower and fruit extracts from *T. lineata* according to their ^{1}H NMR signals**	
Sucrose	δ 5.41 (d, *J*= 3.97 Hz)
	δ 4.18 (d, *J*= 8.5 Hz)
	δ 3.67 (s)
	δ 3.54 (dd, *J*= 9.46 Hz)
	δ 3.54 (dd, *J*= 3.66 Hz)
	δ 4.04 (dd, *J*= 8.24 Hz)
α-Glucose	δ 5.19 (d, *J*= 3.66 Hz)
β -Glucose	δ 4.58 (d, *J*= 7.63 Hz)
Fructose	δ 3.89 (d, *J*= 2.8 Hz)
	δ 3.53 (d, *J*= 11.7 Hz)
Threonine	δ 3.58 (d, *J*= 6.41Hz)
	δ 4.24 (m)
Alanine	δ 1.48 (d, *J*= 7.32 Hz)
	δ 3.8 (m)
Valine	δ 0.95 (d, *J*= 6.71 Hz)
	δ 1.02 (d, *J*= 6.1 Hz)
	δ 3.62 (d, *J*= 4.27 Hz)
Tyrosine	δ 7.18 (t, *J*= 8.5 Hz)
Gamma-aminobutyric acid (GABA)	δ 2.30 (t, *J*= 7.32 Hz)
	δ 3.02 (t, *J*= 7.6 Hz)

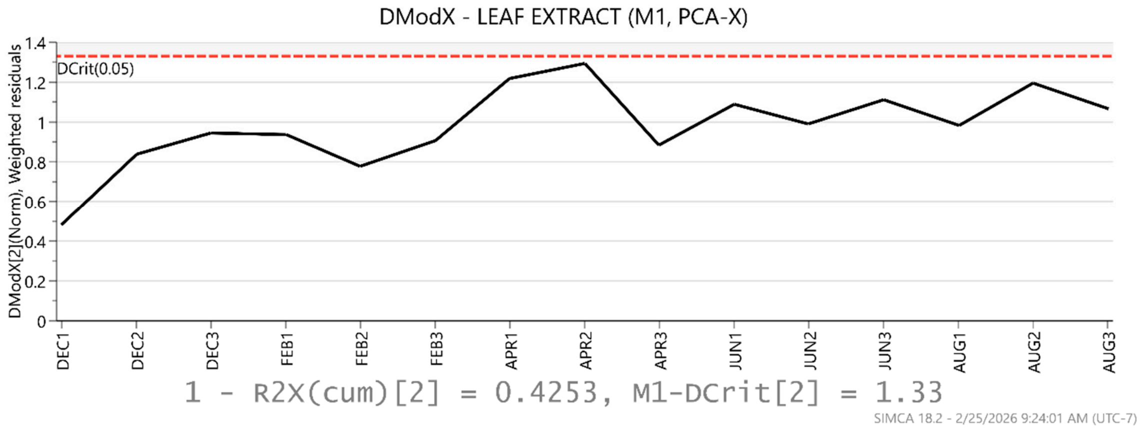

Figure S1. DModX plot for the PCA-X model of the leaf extracts from each month.

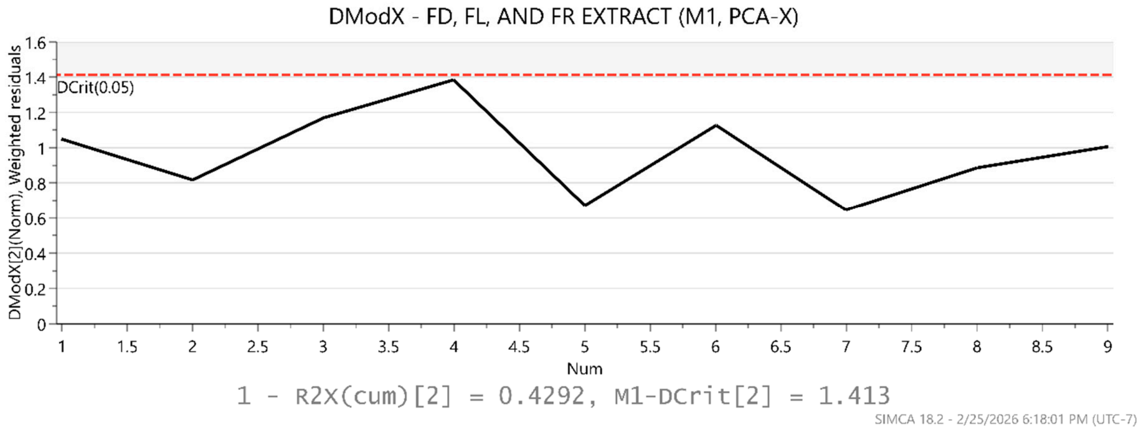

Figure S2. DModX plot for the PCA-X model of the floral bud (FD), flower (FL), and fruit (FR) extracts.
